# Associations of Muscle‐Related Metrics With Respiratory Disease in Chinese Adults: A Prospective Cohort Study

**DOI:** 10.1002/jcsm.13650

**Published:** 2024-11-23

**Authors:** Yongbing Lan, Yalei Ke, Dianjianyi Sun, Pei Pei, Ling Yang, Yiping Chen, Huaidong Du, Silu Lv, Maxim Barnard, Junshi Chen, Zhengming Chen, Jun Lv, Liming Li, Canqing Yu, China Kadoorie Biobank Collaborative Group

**Affiliations:** ^1^ Department of Epidemiology and Biostatistics, School of Public Health Peking University Beijing China; ^2^ Peking University Center for Public Health and Epidemic Preparedness and Response Beijing China; ^3^ Key Laboratory of Epidemiology of Major Diseases (Peking University), Ministry of Education Beijing China; ^4^ Clinical Trial Service Unit and Epidemiological Studies Unit (CTSU), Nuffield Department of Population Health University of Oxford Oxford UK; ^5^ Licang Center for Disease Control and Prevention Qingdao China; ^6^ China National Center for Food Safety Risk Assessment Beijing China; ^7^ State Key Laboratory of Vascular Homeostasis and Remodeling Peking University Beijing China

**Keywords:** grip strength, muscle mass, muscle quality, prospective, respiratory disease

## Abstract

**Background:**

There is limited evidence about the association of muscle mass, strength and quality with respiratory disease, especially in Chinese populations. We aimed to comprehensively examine such associations and identify better metrics with more clinical and public health relevance.

**Methods:**

We conducted a prospective cohort study based on data from the second resurvey of the China Kadoorie Biobank (CKB) study in participants with no prevalent respiratory disease or cancer. Arm muscle quality was calculated as the ratio of grip strength to arm muscle mass. Low muscle mass, grip strength and arm muscle quality were defined as the sex‐specific lowest quintiles of corresponding variables. Cox proportional hazard models were used to estimate the hazard ratios (HRs) and 95% confidence intervals (CIs) for respiratory disease.

**Results:**

In total, 17 510 participants aged 38–88 (65.4% women; mean age 57.8 ± 9.6) were enrolled in 2013–2014 and followed up until 31 December 2018. During a median follow‐up of 4.82 years, 1346 participants developed respiratory disease. After adjustment for sociodemographic characteristics, lifestyle factors and medical histories, the elevated HR of respiratory disease was 1.31 (1.14–1.51) for low grip strength and 1.25 (1.09–1.44) for low arm muscle quality. Grip strength and arm muscle quality exhibited a linearly inverse association between respiratory disease (*p* = 0.137 and 0.102), with each standard deviation (SD) decrease in grip strength and arm muscle quality associated with a 22% (95% CI: 11%–34%) and 14% (95% CI: 7%–22%) increased risk of respiratory disease. No association was found for low total muscle mass index and low appendicular muscle mass index.

**Conclusion:**

Low grip strength and arm muscle quality are associated with increased risks of respiratory disease, and they are better muscle‐related metrics for identifying adults at high risk of respiratory disease. Chinese adults may need to maintain normal muscle mass, strength and quality to achieve better respiratory health, but this needs to be validated in appropriately designed clinical trials.

## 1 Introduction

Sarcopenia is an age‐related progressive skeletal muscle condition characterised by the accelerated decline of muscle mass, muscle strength and physical performance [1]. With increasing life expectancy, the prevalence of sarcopenia in Chinese adults aged ≥ 65 years was 15.0% between 2007 and 2010 and continues to increase [2]. Previous studies have demonstrated that sarcopenia is associated with a significantly increased risk of adverse outcomes such as falls [3], mortality [4], cardiovascular disease (CVD) [5] and respiratory disease [6]. Thus, as important indicators of sarcopenia, low muscle mass and strength may also increase the risk of respiratory disease.

Most previous studies focused on the prevalence and clinical impact of sarcopenia in chronic obstructive pulmonary disease (COPD) or chronic respiratory disease [6, 7], on account of the high prevalence of skeletal muscle dysfunction among corresponding patients, with limited studies that evaluated the relationship between sarcopenia and incident of respiratory disease [8]. Given systemic inflammation, physical inactivity and nutritional deficiencies were considered as part of underlying mechanisms in both sarcopenia and respiratory disease [7, 9, 10], a causal role for sarcopenia was plausible. Moreover, findings on the association between grip strength and respiratory disease were inconsistent. In contrast, no significant association was found between grip strength with respiratory disease and COPD in the Prospective Urban Rural Epidemiology (PURE) study [11]; the study conducted in the UK Biobank reported that grip strength was inversely associated with all respiratory diseases and COPD [12]. Additionally, it is argued that muscle quality, defined as muscle strength per unit of muscle mass [13], is a more sensitive marker of muscle function than muscle mass and strength individually [14]. One study involving the association of muscle‐related indicators with all‐cause mortality demonstrated that muscle strength, such as grip strength and muscle quality, may be better predictors than muscle mass in mortality [15]; it would be interesting to investigate whether the identical phenomenon occurs in respiratory disease. Furthermore, prior studies were mainly conducted in Western populations, with little reliable evidence from Asian populations, especially the Chinese populations [8, 12]. However, Chinese adults' body composition and muscle strength differed substantially from Western populations. Chinese adults have lower lean mass index and lower grip strength when compared with Western adults [16, 17].

This study aims to comprehensively examine the association of muscle mass, grip strength and muscle quality with respiratory disease and identify better metrics with more clinical and public health relevance based on a population‐based prospective cohort, the China Kadoorie Biobank (CKB) study.

## 2 Method

### 2.1 Study Population

The CKB design and implementation details have been described previously [18, 19]. Briefly, 512 724 participants aged 30–79 were enrolled from five urban and five rural areas across China between June 2004 and July 2008 and followed up for nearly 15 years by 31 December 2018. After the baseline survey, periodic resurveys were conducted in 2008, 2013–2014 and 2020–2021 in randomly selected subsamples of about 5% of surviving participants. The present analysis was based on the second resurvey, in which data on muscle mass and grip strength were available. The second resurvey was conducted among 25 239 adults aged 38 to 88 in 2013–2014. All participants provided written informed consent. Ethical approval was obtained from the Ethics Review Committee of the Chinese Center for Disease Control and Prevention (Beijing, China), the Peking University Health Science Center (Beijing, China) and the Oxford Tropical Research Ethics Committee, University of Oxford (UK).

We excluded those with missing data on muscle mass (*n* = 390) or strength (*n* = 294) or physical activities (*n* = 22) and participants who reported a history of respiratory disease (*n* = 6829). In addition, we excluded participants with prevalent cancer (*n* = 194), who may suffer from involuntary weight loss and change their lifestyle. Finally, a total of 17 510 participants were included in the present analysis.

### 2.2 Exposure Assessment

Fat mass and fat‐free mass were measured by Tanita BC418MA (Tanita Inc, Tokyo, Japan) in participants with bare feet and subtracting the corresponding weight of the clothes based on the season and temperature, using the method of bioelectrical impedance analysis (BIA). The accuracy of BIA has been validated [20, 21]. Various muscle mass indices were derived from muscle mass (kg) divided by the square of height (m^2^), including total muscle mass index, appendicular muscle mass index, arm muscle mass index, leg muscle mass index and trunk muscle mass index [22]. Grip strength was measured using a Jamar J00105 hydraulic hand dynamometer (Sammons Preston, Bolingbrook, IL, USA). The mean value of the right and left hands was utilised in the subsequent analyses. Arm muscle quality was calculated as the ratio of grip strength (kg) to arm muscle mass (kg).

According to the Asian Working Group for Sarcopenia (AWGS) [1], the cutoff points used to define low total muscle mass index were the lowest sex‐specific quintiles of total muscle mass index (< 7.46 kg/m^2^ for men and < 6.26 kg/m^2^ for women), and the rest of the participants were defined as having ‘normal’ total muscle mass index. Analogously, low appendicular muscle mass index (< 17.07 kg/m^2^ for men and < 14.76 kg/m^2^ for women), low muscle mass indices by body parts, low grip strength (< 26.5 kg for men and < 16.0 kg for women) and low arm muscle quality (< 10.23 kg/kg for men and < 8.89 kg/kg for women) were defined as the lowest quintiles of the corresponding variables.

### 2.3 Assessment of Covariates

Information at the second resurvey was collected by trained staff, including an interviewer‐administered questionnaire, physical measurements and blood sample collection. The questionnaire inquired about information on sociodemographic characteristics (age, gender, urban or rural residence, educational attainment, household income and marital status), lifestyle factors (tobacco smoking, passive smoking, alcohol consumption, dietary habits, household air pollution and physical activity) and personal medical history. Additionally, trained technicians measured body fat percentage, height, weight, waist circumference (WC), blood pressure, random plasma glucose and lung function with calibrated instruments and standard protocols. Prevalent CVD was defined as a self‐reported diagnosis of coronary heart disease (CHD), stroke, acute myocardial infarction (AMI), angina or other ischemic heart disease. Prevalent diabetes was defined as a measured fasting blood glucose concentration > 7.0 mmol/L, a measured random blood glucose concentration > 11.1 mmol/L or a self‐reported diagnosis of diabetes. Prevalent COPD was defined as the ratio of the forced expiratory volume in 1 s over force vital capacity [(FEV_1_/FVC) < 0.7] or a self‐reported diagnosis of COPD, chronic bronchitis or emphysema. Prevalent respiratory disease was defined as prevalent COPD or self‐reported asthma, tuberculosis or pulmonary heart disease diagnosis.

### 2.4 Assessment of Outcome

Information on respiratory disease morbidity was identified through electronic linkage with the Chinese Disease Surveillance Points (DSP) system and the National Health Insurance System. The former provides vital status for the entire country [23], and the latter covers more than 97% of the participants in the CKB study. In addition, the follow‐up of the CKB study was supplemented with official residential records and annual active follow‐up. All events were coded by trained staff blinded to baseline information according to the International Classification of Diseases, 10th Revision (ICD‐10). Respiratory diseases were defined as ICD‐10 J09‐J98 and I26‐I27 recorded on hospital admission or death records. The follow‐up duration was calculated from the date of the second resurvey to the date of respiratory disease incidence (*n* = 1346), death (*n* = 475), lost to follow‐up (*n* = 26), or 31 December 2018 (*n* = 15 663), whichever came first.

### 2.5 Statistical Analysis

Characteristics of the participants were described as mean for continuous variables using linear regression models and percentages for categorical variables using logistic models, adjusted for age, sex and study area, where appropriate.

In the prospective analysis of low muscle mass indices, grip strength and arm muscle quality with respiratory disease, we first used Cox proportional hazards models to estimate the hazard ratios (HRs) and 95% confidence intervals (CIs) with age as the time scale. We stratified by age at the study date in 5‐year intervals, sex and study region. Potential confounders were adjusted for in the multivariate Cox model: education attainment, household income, marital status, smoking status, passive smoking, cooking and heating fuel type, alcohol drinking, intake frequencies of red meat, fresh fruit and vegetables, BMI, WC, physical activities, prevalent CVD and diabetes. A directed acyclic graph explaining the association between the exposures, the outcome and covariates was available (Figure S1). The proportional hazard assumption was checked via Schoenfeld residuals, and all of the *p*‐values were more than 0.05. Additionally, we used restricted cubic splines with four knots at the 5th, 35th, 65th and 95th percentages of grip strength or arm muscle mass quality to examine the non‐linear relationship. If there were linear correlations of grip strength and arm muscle quality with respiratory disease, quintiles of grip strength and arm muscle quality would enter the model, with the third quintile being the reference group to calculate relative risks. Several sensitivity analyses were conducted to test the robustness of our results: additionally excluding participants with self‐reported CVD or diabetes, restricting the analysis to the never‐smokers, excluding participants who developed respiratory disease in the first year of follow‐up to minimise reverse causation, additionally adjusted for smoking duration, using Fine–Gray competing risks regression, and defining muscle quality as the ratio of grip strength to total muscle mass.

We further conducted subgroup analysis to assess the heterogeneity of the association across sex (male or female), age (< 60 or ≥ 60 years), region (urban or rural area), levels of physical activities (high or low), BMI (< 24 or ≥ 24 kg/m^2^) and WC (male/female: < 85/< 80 or ≥ 85/≥ 80 cm). The potential multiplicative interactions were tested using likelihood ratio tests by comparing models with and without the interaction terms.

The forest plot was drawn by R (version 4.3.1), and all other statistical analyses were performed using Stata 15.0 (StataCorp, College Station, TX, USA). All *p*‐values were two‐sided with a significance threshold of 0.05.

## 3 Result

Among the 17 510 eligible participants, 11 449 (65.4%) were female, and 9637 (55.0%) were from rural areas. The mean age at the second resurvey was 57.8 ± 9.6 years. During a median follow‐up period of 4.82 years (interquartile range: 4.53–5.14 years), 1346 participants developed respiratory disease. Compared with adults with normal total muscle mass index, those with low total muscle mass index tended to be female; older; resided in urban areas; were physically inactive; had lower BMI, body fat percentage and WC; and were less likely to report prevalent diabetes (Table 1).

**TABLE 1:** Characteristics of participants by total muscle mass index.

Characteristics	Low^a^	Normal^a^	
Total *n*	3504	14 006	*p*
Sociodemographic			
Female, %	2291 (66.3)	9158 (65.2)	0.008
Age, years	60.3 ± 10.3	57.1 ± 9.4	< 0.001
Urban area, %	1845 (51.6)	6028 (43.3)	< 0.001
Married, %	3021 (88.2)	12 552 (89.1)	0.135
Middle school and higher, %	1599 (53.0)	7381 (50.9)	0.782
Income ≥ 20 000 yuan/year, %	1574 (38.2)	5306 (39.6)	0.385
Lifestyle factors			
Current daily smoker, %	745 (21.9)	2759 (19.8)	0.133
Passive smoker, %	1826 (54.5)	7791 (55.0)	0.894
Current daily drinker, %	285 (7.9)	1069 (7.7)	0.952
Consuming meat daily, %	1457 (42.1)	6020 (42.9)	0.874
Consuming fresh vegetables daily, %	3391 (96.9)	13 593 (97.0)	0.232
Consuming fresh fruit daily, %	1134 (34.6)	5062 (35.6)	0.792
Cooking with solid fuel, %	848 (26.9)	3786 (26.4)	0.091
Heating with solid fuel, %	589 (23.6)	3564 (23.7)	0.393
Physical activity, MET h/day	18.1 ± 13.6	19.1 ± 13.7	< 0.001
Anthropometric measures			
BMI, kg/m^2^	20.8 ± 2.1	25.4 ± 3.1	< 0.001
Body fat percentage, %	23.7 ± 8.0	28.1 ± 8.1	< 0.001
Waist circumference, cm	76.5 ± 7.6	86.9 ± 9.2	< 0.001
Grip strength, kg	23.5 ± 8.1	25.4 ± 9.2	< 0.001
Health status			
History of CVD, %	238 (6.7)	1169 (8.4)	0.161
History of diabetes, %	247 (6.3)	1452 (10.7)	0.007

*Note:* Characteristics were adjusted for sex, age and study area as appropriate.

Abbreviations: AWGS, Asian Working Group for Sarcopenia; BMI, body mass index; *n*, number; CVD, cardiovascular disease; MET, metabolic equivalent of task.

^a^ According to the AWGS, low total muscle mass index was defined as the lowest sex‐specific quintiles of total muscle mass index. Normal groups were defined as Q2–Q5 of total muscle mass index.

### 3.1 Muscle Mass and Respiratory Disease Morbidity

The nonfully adjusted HR of respiratory disease was 1.20 (95% CI: 1.04–1.37) for low total muscle mass index compared with normal total muscle mass index and no association between appendicular muscle mass index and respiratory disease incidence (Table 2), but the association of total muscle mass index with respiratory disease was no longer significant when further adjusted for lifestyle factors and medical histories.

**TABLE 2:** Association of muscle mass indices with respiratory disease.

Muscle mass indices	Binary category	
	Normal^a^	Low^a^
Total muscle mass index		
Cases, *n*	1023	323
Rate^b^	16.18	19.34
Model 1	1.00	1.20 (1.04, 1.37)
Model 2	1.00	1.13 (0.96, 1.34)
Model 3	1.00	1.14 (0.96, 1.34)
Appendicular muscle mass index		
Cases, *n*	1013	333
Rate^b^	16.42	18.28
Model 1	1.00	1.12 (0.98, 1.28)
Model 2	1.00	1.04 (0.88, 1.23)
Model 3	1.00	1.05 (0.89, 1.24)
Arm muscle mass index		
Cases, *n*	1011	335
Rate^b^	16.08	19.65
Model 1	1.00	1.21 (1.05, 1.39)
Model 2	1.00	1.16 (0.98, 1.36)
Model 3	1.00	1.16 (0.98, 1.36)
Leg muscle mass index		
Cases, *n*	1007	339
Rate^b^	16.41	18.26
Model 1	1.00	1.13 (0.99, 1.29)
Model 2	1.00	1.07 (0.91, 1.25)
Model 3	1.00	1.07 (0.91, 1.26)
Trunk muscle mass index		
Cases, *n*	1052	294
Rate^b^	16.28	19.26
Model 1	1.00	1.19 (1.03, 1.39)
Model 2	1.00	1.14 (0.96, 1.35)
Model 3	1.00	1.14 (0.96, 1.35)

*Note:* HRs were stratified by sex, age and study regions and shown as point estimate value (95% confidence interval); Model 1 was adjusted for education, household income and marital status; Model 2 was additionally adjusted alcohol consumption, tobacco smoking, BMI, waist circumference, fresh fruit consumption, fresh vegetables consumption, red meat consumption, passive smoking, physical activity, cook pollution and heat pollution; Model 3 was additionally adjusted for history of cardiovascular diseases and diabetes.

Abbreviation: HRs, hazard ratios.

^a^ According to the AWGS, low muscle mass indices were defined as the lowest sex‐specific quintiles of corresponding muscle mass indices, respectively. Normal groups were defined as Q2–Q5 of corresponding muscle mass indices, respectively.

^b^ Per 1000 person‐years.

In the association between low muscle mass indices and respiratory disease incidence by body parts (Table 2), the nonfully adjusted HR for low arm muscle mass index was 1.21 (95% CI: 1.05–1.39) compared with the normal group and 1.19 (95% CI: 1.03–1.39) for low trunk muscle mass. The association was not statistically significant after adjusting lifestyle factors and medical histories.

### 3.2 Grip Strength, Muscle Quality and Respiratory Disease Morbidity

**FIGURE 1:**
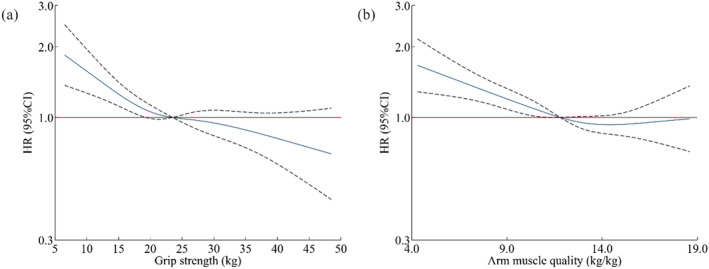
Restricted cubic splines for associations between grip strength and arm muscle quality with respiratory disease. HR: hazard ratio; CI: confidence interval. The four knots for restricted cubic splines were set at the 5th, 35th, 65th and 95th percentages of grip strength (or arm muscle quality), and the median values of corresponding indices were the reference points. Solid lines represent HRs, and dashed lines represent 95% CIs. Models were adjusted for variables in Model 3 of Table 2. The *p*‐values for nonlinearity are as follows: (a) grip strength: 0.137; (b) arm muscle quality: 0.102.

The adjusted HR (95% CI) for the low grip strength group was 1.31 (1.14–1.51) compared with the normal group and 1.25 (1.09–1.44) for the low arm muscle quality group (Table 3). The restricted cubic spline model showed a linear association between grip strength, arm muscle quality and respiratory disease risk (*p* = 0.137 and 0.102, Figure 1).

**TABLE 3:** Association of grip strength and arm muscle quality with respiratory disease.

Muscle mass indices	Binary category	
	Normal^a^	Low^a^
Grip strength		
Cases, *n*	890	456
Rate^b^	15.40	20.46
Model 1	1.00	1.37 (1.19, 1.57)
Model 2	1.00	1.34 (1.16, 1.53)
Model 3	1.00	1.31 (1.14, 1.51)
Arm muscle quality		
Cases, *n*	969	377
Rate^b^	15.95	19.60
Model 1	1.00	1.27 (1.11, 1.46)
Model 2	1.00	1.28 (1.11, 1.47)
Model 3	1.00	1.25 (1.09, 1.44)

*Note:* HRs were stratified by sex, age and study regions and shown as point estimate values (95% confidence interval). Models 1 to 3 were adjusted for the same variables as the corresponding models in Table 2.

Abbreviation: HRs, hazard ratios.

^a^ According to the AWGS, low grip strength and low arm muscle quality were defined as the lowest sex‐specific quintiles of grip strength and arm muscle quality, respectively. Normal groups were defined as Q2–Q5 of grip strength and arm muscle quality, respectively.

^b^ Per 1000 person‐years.

**FIGURE 2:**
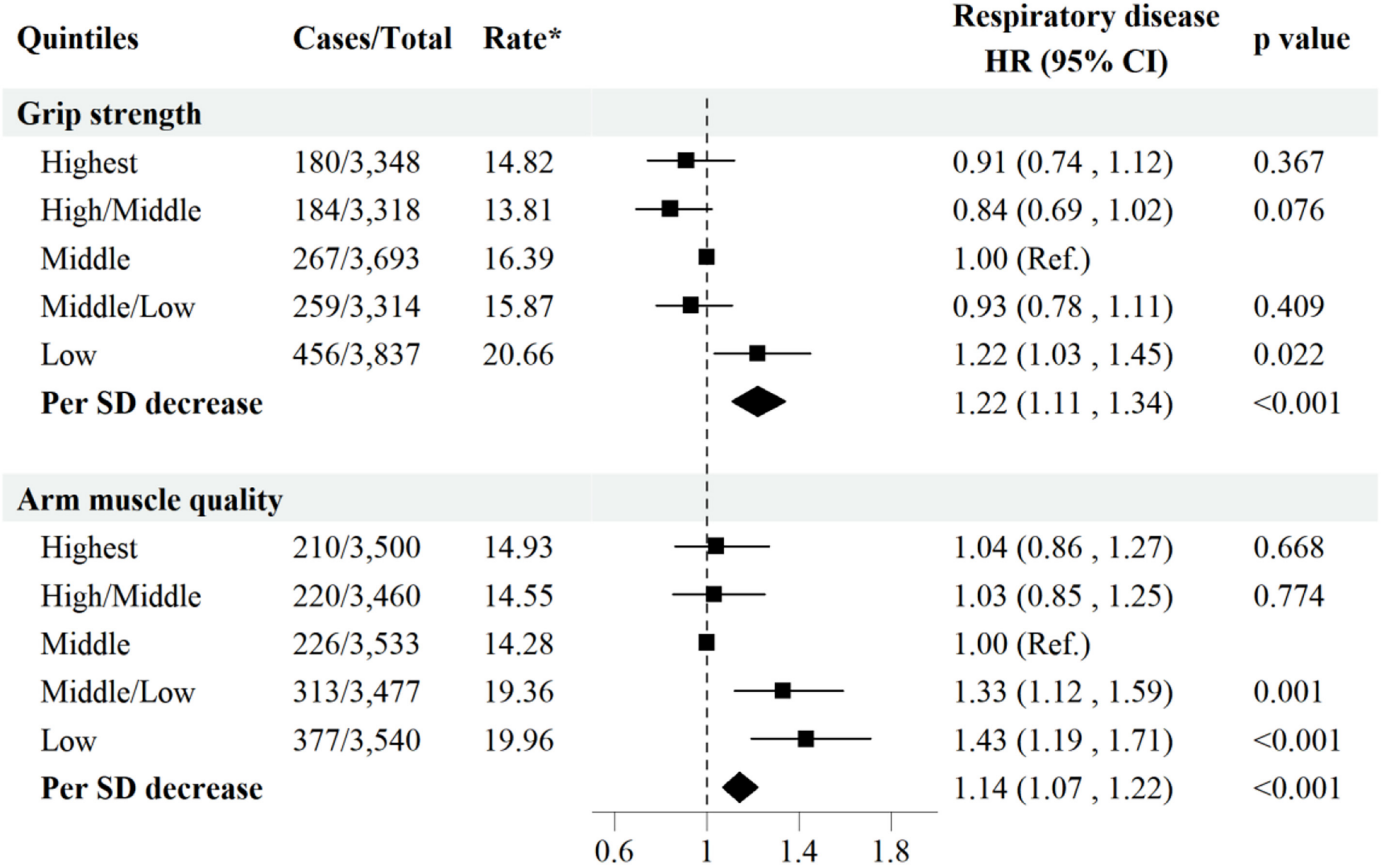
Associations between quintiles of grip strength, arm muscle quality and respiratory disease. HR: hazard ratio; CI: confidence interval. Models were adjusted for variables in Model 3 of Table 2. *Per 1000 person‐years.

Further grouping grip strength and arm muscle quality according to the quintiles of corresponding variables, the relative risks of adults in the lowest quintiles of grip strength and arm muscle quality were 1.22 (1.03–1.45) and 1.43 (1.19–1.71), respectively, when comparing with the third quintile. Moreover, each standard deviation (SD) decrease in grip strength and arm muscle quality was associated with a 22% (95% CI: 11%–34%) and 14% (95% CI: 7%–22%) increased risk of respiratory disease (Figure 2).

### 3.3 Sensitivity Analysis and Stratified Analysis

In sensitivity analysis, the associations between grip strength, arm muscle quality and respiratory disease were not substantially altered when further excluding participants with self‐reported diagnosis of cardiovascular disease or diabetes (*n* = 2842), excluding participants who developed respiratory disease in the first year of follow‐up (*n* = 302), additionally adjusted for smoking duration, using Fine–Gray competing risks regression and defining muscle quality as the ratio of grip strength to total muscle mass (Tables S1 and S3–S6). However, among never‐smokers (*n* = 13 596), we found the association of low total muscle mass index, low arm muscle mass index and low trunk muscle mass with respiratory disease, and the adjusted HRs (95% CI) were 1.30 (1.08–1.57), 1.29 (1.07–1.56) and 1.25 (1.03–1.52), respectively (Table S2).

In the subgroup analysis, no interactions were observed between muscle indices and various subgrouping variables on respiratory disease (all *p*‐values for interaction > 0.05).

## 4 Discussion

In our large population‐based cohort study, the increased risk of respiratory disease was 31% for low grip strength and 25% for low arm muscle quality, while no statistically significant association was found for low total muscle mass or low appendicular muscle mass index. Grip strength and arm muscle quality were linearly inversely associated with respiratory disease incidence, with a per‐SD decrease in grip strength associated with a 22% increased risk of respiratory disease morbidity and a per‐SD decrease in arm muscle quality associated with a 14% increased risk of respiratory disease morbidity.

Although previous studies reported the association of sarcopenia and muscle mass with cardiovascular disease and all‐cause mortality [4, 5], few studies examined the association between sarcopenia, muscle mass and respiratory disease. The UK Biobank study, which involved 170 083 adults with a median follow‐up of 6.1 years, found that sarcopenia and sarcopenia obesity were associated with a 23% (95% CI: 10%–36%) and 51% (95% CI: 30%–77%) higher risk of respiratory disease incidence, respectively [12]. The Specialized Centers of Clinically Oriented Research (SCCOR) followed 246 participants for 6 years and reported that incident low appendicular muscle mass was associated with greater lung diseases [24]. Unlike previous studies, we did not observe the increased risks of low total and appendicular muscle mass with respiratory disease. However, we found that the HR for nonsmokers with low total muscle mass index was 1.30 (95% CI: 1.08–1.57). As a prominent risk factor for respiratory disease [25], smoking may overshadow the effect of low muscle mass on respiratory disease.

Previous cohort studies reported inconsistent results on the relationship between grip strength and respiratory disease [11, 12]. The PURE study enrolled 142 861 participants (including 46 036 Chinese) from 17 countries and reported no association between grip strength and respiratory disease during a median follow‐up of 4.0 years [11], while the UK Biobank study including 502 293 participants aged 40–69 years (54% women) reported that HRs per 5 kg lower grip strength were higher for all respiratory disease (1.31, 1.22–1.40 and 1.24, 1.20–1.28) and COPD (1.24, 1.05–1.47 and 1.19, 1.09–1.30) during a median follow‐up of 7.1 years, in women and men, respectively [12]. Of note, the cross‐sectional analysis conducted in the Genetic Epidemiology of COPD study (COPDGene) revealed that in ever‐smokers with COPD, independent of BMI and emphysema, lower grip strength was associated with higher exacerbation frequency [26]. Similar to findings from the UK Biobank, our study observed that grip strength was inversely associated with the risk of respiratory disease. In addition, we investigated the association between muscle quality and respiratory disease and found that low arm quality was associated with an increased risk of respiratory disease. Previous evidence reported the association of low arm quality with all‐cause mortality among 2292 Americans aged 70–79 [15]. Furthermore, prior research indicated that longitudinal changes in muscle mass and strength were associated with increased all‐cause mortality and CVD [27, 28]. However, the present study only examined the effect of baseline muscle‐related metrics on respiratory health, while our data suggested that the metrics during follow‐up were highly correlated (the Person correlation coefficient ranging from 0.53 to 0.88).

The associations of respiratory disease incidence with low appendicular muscle mass index and low total muscle mass index were only observed in several subgroups and sensitivity analyses. In contrast, the associations with low grip strength and low arm muscle quality were stable after fully adjusting for sociodemographic factors, lifestyle factors and medical histories. This indicates that grip strength and arm muscle quality are more related to clinical and public health and should be given primary consideration when assessing population respiratory disease incidence risk using different muscle metrics.

While the biological mechanism between muscle strength and respiratory disease is yet to be explored, some mechanisms may explain the corresponding results in the present study. Muscle mass and strength are indicators for nutritional status and physical performance [29, 30], and analysis from the Korean Nation Health and Nutrition Examination Surveys has shown that moderate to vigorous physical activities were highly correlated with muscle mass and strength [31]. A previous clinical trial has also demonstrated that combining whey protein, vitamin D and E can significantly improve muscle mass and strength [10]. Thus, lifestyle factors are essential in maintaining muscle mass and strength. While malnutrition and low levels of physical activity are associated with respiratory disease [32, 33], in the present study, we observed the association of muscle mass and strength with respiratory disease after fully adjusting for diet and physical activity levels. What's more, elevated levels of inflammatory cytokine were observed in the serum of people with sarcopenia, negatively correlated with lung function [34].

The present study was conducted among a community‐based population from diverse regions across China and comprehensively examined the association of respiratory disease with several muscle‐related metrics. Nevertheless, some limitations need to be discussed. First, physical performance, such as gait speed, was recommended as a key element by AGWS to define sarcopenia. However, information on these muscle‐related metrics was unavailable in the present study. Second, the muscle mass was measured with the BIA method; its accuracy is less reliable than the dual‐energy X‐ray absorptiometry (DEXA) method. Nevertheless, BIA is relatively inexpensive, noninvasive, portable and convenient [20]. Therefore, it is feasible in the large population‐based field investigations. Third, many covariates were self‐reported, which may suffer from measurement error. However, most measurements, such as smoking status, showed good reproducibility. Fourth, the duration of follow‐up was relatively shorter, whereas the development of chronic respiratory disease was a long‐term process. Thus, studies with longer follow‐up periods were warranted to validate our results. Fifth, unmeasured or residual confounding was possible, such as serum 25‐hydroxyvitamin D (25(OH)D), which played an important role in muscle function and respiratory disease [35, 36]. Finally, due to the observational nature of the present study, the potential causal association should be confirmed in future intervention trials.

## 5 Conclusion

Low grip strength and low arm muscle quality are independent risk factors for respiratory disease incidence among Chinese adults. The association between low muscle mass and respiratory diseases is inconsistent, indicating that grip strength and arm muscle quality are better indicators of respiratory disease prediction. Meanwhile, improving muscle mass, strength and quality levels may benefit respiratory health in Chinese adults.

## Conflicts of Interest

The authors declare no conflicts of interest.

## Supporting information


**Figure S1.** Directed acyclic graph (DAG) explaining the association between the exposures, the outcome and covariates included in the analyses.
**Table S1.** Sensitivity analyses for associations of muscle mass indices, grip strength and arm muscle quality with respiratory disease (Further excluding participants with self‐reported diagnoses of cardiovascular disease or diabetes).
**Table S2.** Sensitivity analyses for associations of muscle mass indices, grip strength and arm muscle quality with respiratory disease (Excluding participants who have ever smoked).
**Table S3.** Sensitivity analyses for associations of muscle mass indices, grip strength and arm muscle quality with respiratory disease (Excluding participants who developed respiratory disease in the first year of follow‐up).
**Table S4.** Sensitivity analyses for associations of muscle mass indices, grip strength and arm muscle quality with respiratory disease (additionally adjusted for smoking duration1).
**Table S5.** Sensitivity analyses for associations of muscle mass indices, grip strength and arm muscle quality with respiratory disease (using Fine–Gray competing risks regression).
**Table S6.** Associations of muscle quality with respiratory disease.
**Table S7.** Associations of muscle mass indices, grip strength and arm muscle quality with respiratory disease by sex.
**Table S8.** Associations of muscle mass indices, grip strength and arm muscle quality with respiratory disease by age.
**Table S9.** Associations of muscle mass indices, grip strength and arm muscle quality with respiratory disease by study areas.
**Table S10.** Associations of muscle mass indices, grip strength and arm muscle quality with respiratory disease by levels of physical activities.
**Table S11.** Associations of muscle mass indices, grip strength and arm muscle quality with respiratory disease by BMI.
**Table S12.** Associations of muscle mass indices, grip strength and arm muscle quality with respiratory disease by waist circumference (WC).

## Data Availability

Details of how to access China Kadoorie Biobank data and details of the data release schedule are available online (www.ckbiobank.org/site/Data+Access).
