# Supplementary material for: Associations of Muscle‐Related Metrics With Respiratory Disease in Chinese Adults: A Prospective Cohort Study
Source: J Cachexia Sarcopenia Muscle. 2024 Nov 23;16(1):e13650. doi: 10.1002/jcsm.13650 (PMC11695270; doi:10.1002/jcsm.13650)
Supplement: Supplementary file 1 — Figure S1. Directed acyclic graph (DAG) explaining the association between the exposures, the outcome and covariates included in the analyses. Table S1. Sensitivity analyses for associations of muscle mass indices, grip strength and arm muscle quality with respiratory disease (Further excluding participants with self‐reported diagnoses of cardiovascular disease or diabetes). Table S2. Sensitivity analyses for associations of muscle mass indices, grip strength and arm muscle quality with respiratory disease (Excluding participants who have ever smoked). Table S3. Sensitivity analyses for associations of muscle mass indices, grip strength and arm muscle quality with respiratory disease (Excluding participants who developed respiratory disease in the first year of follow‐up). Table S4. Sensitivity analyses for associations of muscle mass indices, grip strength and arm muscle quality with respiratory disease (additionally adjusted for smoking duration1). Table S5. Sensitivity analyses for associations of muscle mass indices, grip strength and arm muscle quality with respiratory disease (using Fine–Gray competing risks regression). Table S6. Associations of muscle quality with respiratory disease. Table S7. Associations of muscle mass indices, grip strength and arm muscle quality with respiratory disease by sex. Table S8. Associations of muscle mass indices, grip strength and arm muscle quality with respiratory disease by age. Table S9. Associations of muscle mass indices, grip strength and arm muscle quality with respiratory disease by study areas. Table S10. Associations of muscle mass indices, grip strength and arm muscle quality with respiratory disease by levels of physical activities. Table S11. Associations of muscle mass indices, grip strength and arm muscle quality with respiratory disease by BMI. Table S12. Associations of muscle mass indices, grip strength and arm muscle quality with respiratory disease by waist circumference (WC). [file JCSM-16-e13650-s001.docx]

# Associations of Muscle-related Metrics with Respiratory Disease in Chinese Adults: A Prospective Cohort Study

**Supplementary Material**

[Figure S1 Directed acyclic graph (DAG) explaining the association between the exposures, the outcome, and covariates included in the analyses. 3](#_Toc178767256)

[Table S1 Sensitivity analyses for associations of muscle mass indices, grip strength, and arm muscle quality with respiratory disease (Further excluding participants with self-reported diagnoses of cardiovascular disease or diabetes). 4](#_Toc178767257)

[Table S2 Sensitivity analyses for associations of muscle mass indices, grip strength, and arm muscle quality with respiratory disease (Excluding participants who have ever smoked). 6](#_Toc178767258)

[Table S3 Sensitivity analyses for associations of muscle mass indices, grip strength, and arm muscle quality with respiratory disease (Excluding participants who developed respiratory disease in the first year of follow-up). 7](#_Toc178767259)

[Table S4 Sensitivity analyses for associations of muscle mass indices, grip strength, and arm muscle quality with respiratory disease (additionally adjusted for smoking duration^1^). 8](#_Toc178767260)

[Table S5 Sensitivity analyses for associations of muscle mass indices, grip strength, and arm muscle quality with respiratory disease (using Fine-Gray competing risks regression). 8](#_Toc178767261)

[Table S6 Associations of muscle quality with respiratory disease. 9](#_Toc178767262)

[Table S7 Associations of muscle mass indices, grip strength, and arm muscle quality with respiratory disease by sex. 10](#_Toc178767263)

[Table S8 Associations of muscle mass indices, grip strength, and arm muscle quality with respiratory disease by age. 11](#_Toc178767264)

[Table S9 Associations of muscle mass indices, grip strength, and arm muscle quality with respiratory disease by study areas. 12](#_Toc178767265)

[Table S10 Associations of muscle mass indices, grip strength, and arm muscle quality with respiratory disease by levels of physical activities. 13](#_Toc178767266)

[Table S11 Associations of muscle mass indices, grip strength, and arm muscle quality with respiratory disease by BMI. 14](#_Toc178767267)

[Table S12 Associations of muscle mass indices, grip strength, and arm muscle quality with respiratory disease by waist circumference (WC). 15](#_Toc178767268)


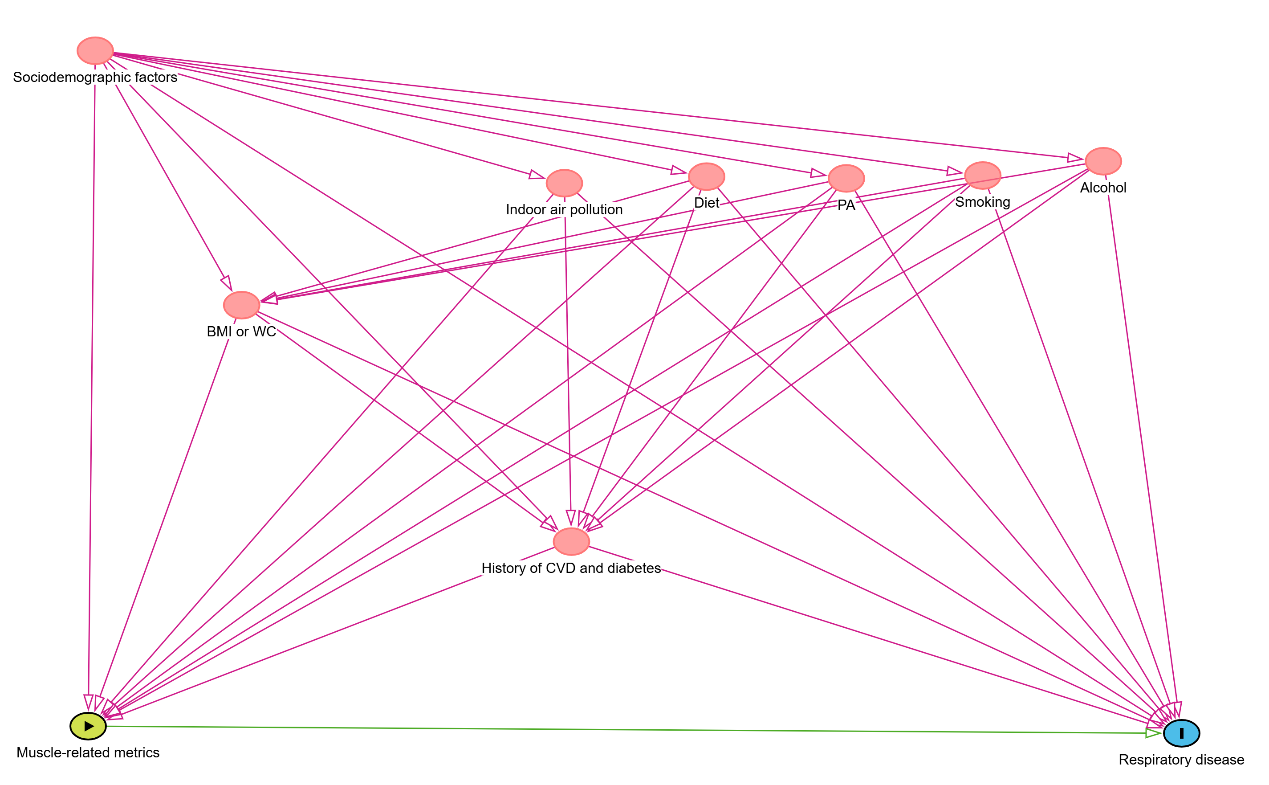


# Figure S1 Directed acyclic graph (DAG) explaining the association between the exposures, the outcome, and covariates included in the analyses.

Sociodemographic factors included age, sex, study regions, education, household income, and marital status; muscle-related metrics included muscle mass, grip strength, and muscle quality; diet included consumption of red meat, fresh fruit and vegetables; indoor air pollution included passive smoking, cook pollution, and heat pollution.

PA, physical activity; BMI, body mass index; WC, waist circumference; CVD, cardiovascular disease.

DAG was drawn using [https://www.dagitty.net/](https://dagitty.net/dags.html)

# Table S1 Sensitivity analyses for associations of muscle mass indices, grip strength, and arm muscle quality with respiratory disease (Further excluding participants with self-reported diagnoses of cardiovascular disease or diabetes).

| **Muscle mass indices** | **Binary category** | |
| --- | --- | --- |
|  | **Normal**^1^ | **Low**^1^ |
| **Total muscle mass index** | | |
| Model 1 | 1.00 | 1.23(1.06,1.43) |
| Model 2 | 1.00 | 1.17(0.97,1.40) |
| **Appendicular muscle mass indx** | | |
| Model 1 | 1.00 | 1.11(0.95,1.29) |
| Model 2 | 1.00 | 1.02(0.85,1.22) |
| **Arm muscle mass index** | | |
| Model 1 | 1.00 | 1.24(1.06,1.45) |
| Model 2 | 1.00 | 1.18(0.99,1.42) |
| **Leg muscle mass index** | | |
| Model 1 | 1.00 | 1.14(0.98,1.32) |
| Model 2 | 1.00 | 1.07(0.89,1.28) |
| **Trunk muscle mass index** | | |
| Model 1 | 1.00 | 1.19(1.01,1.42) |
| Model 2 | 1.00 | 1.13(0.93,1.36) |
| **Grip strength** | | |
| Model 1 | 1.00 | 1.29(1.10,1.51) |
| Model 2 | 1.00 | 1.25(1.07,1.47) |
| **Arm muscle quality** | | |
| Model 1 | 1.00 | 1.22(1.04,1.44) |
| Model 2 | 1.00 | 1.26(1.07,1.49) |

HRs were stratified by sex, age, and study regions and shown as point estimate values (95% confidence interval). Models 1 to 2 were adjusted for the same variables as the corresponding models in Table 2. ^1^According to the AWGS, low muscle mass indices, low grip strength and low arm muscle quality were defined as the lowest sex-specific quintiles of corresponding muscle mass indices, grip strength and arm muscle quality, respectively. Normal groups were defined as Q2-Q5 of corresponding muscle mass indices, grip strength and arm muscle quality, respectively. HRs, hazard ratios.

# Table S2 Sensitivity analyses for associations of muscle mass indices, grip strength, and arm muscle quality with respiratory disease (Excluding participants who have ever smoked).

| **Muscle mass indices** | **Binary category** | |
| --- | --- | --- |
|  | **Normal**^1^ | **Low**^1^ |
| **Total muscle mass index** | | |
| Model 1 | 1.00 | 1.33(1.14,1.55) |
| Model 2 | 1.00 | 1.30(1.08,1.57) |
| Model 3 | 1.00 | 1.30(1.08,1.57) |
| **Appendicular muscle mass index** | | |
| Model 1 | 1.00 | 1.21(1.04,1.41) |
| Model 2 | 1.00 | 1.15(0.96,1.38) |
| Model 3 | 1.00 | 1.16(0.96,1.39) |
| **Arm muscle mass index** | | |
| Model 1 | 1.00 | 1.32(1.13,1.54) |
| Model 2 | 1.00 | 1.29(1.07,1.56) |
| Model 3 | 1.00 | 1.29(1.07,1.56) |
| **Leg muscle mass index** | | |
| Model 1 | 1.00 | 1.19(1.02,1.39) |
| Model 2 | 1.00 | 1.13(0.95,1.36) |
| Model 3 | 1.00 | 1.14(0.95,1.36) |
| **Trunk muscle mass index** | | |
| Model 1 | 1.00 | 1.31(1.10,1.56) |
| Model 2 | 1.00 | 1.25(1.03,1.51) |
| Model 3 | 1.00 | 1.25(1.03,1.52) |
| **Grip strength** | | |
| Model 1 | 1.00 | 1.37(1.17,1.60) |
| Model 2 | 1.00 | 1.35(1.16,1.58) |
| Model 3 | 1.00 | 1.34(1.15,1.57) |
| **Arm muscle quality** | | |
| Model 1 | 1.00 | 1.21(1.03,1.41) |
| Model 2 | 1.00 | 1.21(1.03,1.42) |
| Model 3 | 1.00 | 1.20(1.02,1.40) |

HRs were stratified by sex, age, and study regions and shown as point estimate values (95% confidence interval). Models 1 to 3 were adjusted for the same variables as the corresponding models in Table 2. ^1^According to the AWGS, low muscle mass indices, low grip strength and low arm muscle quality were defined as the lowest sex-specific quintiles of corresponding muscle mass indices, grip strength and arm muscle quality, respectively. Normal groups were defined as Q2-Q5 of corresponding muscle mass indices, grip strength and arm muscle quality, respectively. HRs, hazard ratios.

# Table S3 Sensitivity analyses for associations of muscle mass indices, grip strength, and arm muscle quality with respiratory disease (Excluding participants who developed respiratory disease in the first year of follow-up).

| **Muscle mass indices** | **Binary category** | |
| --- | --- | --- |
|  | **Normal**^1^ | **Low**^1^ |
| **Total muscle mass index** | | |
| Model 1 | 1.00 | 1.12(0.96,1.30) |
| Model 2 | 1.00 | 1.07(0.89,1.29) |
| Model 3 | 1.00 | 1.07(0.89,1.29) |
| **Appendicular muscle mass index** | | |
| Model 1 | 1.00 | 1.09(0.94,1.26) |
| Model 2 | 1.00 | 1.03(0.86,1.24) |
| Model 3 | 1.00 | 1.04(0.87,1.25) |
| **Arm muscle mass index** | | |
| Model 1 | 1.00 | 1.16(1.00,1.35) |
| Model 2 | 1.00 | 1.13(0.94,1.36) |
| Model 3 | 1.00 | 1.13(0.94,1.35) |
| **Leg muscle mass index** | | |
| Model 1 | 1.00 | 1.12(0.97,1.30) |
| Model 2 | 1.00 | 1.09(0.91,1.30) |
| Model 3 | 1.00 | 1.09(0.91,1.30) |
| **Trunk muscle mass index** | | |
| Model 1 | 1.00 | 1.15(0.97,1.36) |
| Model 2 | 1.00 | 1.13(0.94,1.37) |
| Model 3 | 1.00 | 1.13(0.94,1.37) |
| **Grip strength** | | |
| Model 1 | 1.00 | 1.32(1.14,1.54) |
| Model 2 | 1.00 | 1.30(1.11,1.51) |
| Model 3 | 1.00 | 1.27(1.09,1.48) |
| **Arm muscle quality** | | |
| Model 1 | 1.00 | 1.30(1.12,1.51) |
| Model 2 | 1.00 | 1.30(1.11,1.51) |
| Model 3 | 1.00 | 1.26(1.09,1.47) |

HRs were stratified by sex, age, and study regions and shown as point estimate values (95% confidence interval). Models 1 to 3 were adjusted for the same variables as the corresponding models in Table 2, except for tobacco smoking. ^1^According to the AWGS, low muscle mass indices, low grip strength and low arm muscle quality were defined as the lowest sex-specific quintiles of corresponding muscle mass indices, grip strength and arm muscle quality, respectively. Normal groups were defined as Q2-Q5 of corresponding muscle mass indices, grip strength and arm muscle quality, respectively. HRs, hazard ratios.

# Table S4 Sensitivity analyses for associations of muscle mass indices, grip strength, and arm muscle quality with respiratory disease (additionally adjusted for smoking duration^1^).

| **Muscle mass indices** | **Binary category** | |
| --- | --- | --- |
|  | **Normal**^2^ | **Low**^2^ |
| Total muscle mass index | 1.00 | 1.14(0.96,1.34) |
| Appendicular muscle mass index | 1.00 | 1.05(0.89,1.24) |
| Arm muscle mass index | 1.00 | 1.16(0.98,1.37) |
| Leg muscle mass index | 1.00 | 1.07(0.91,1.26) |
| Trunk muscle mass index | 1.00 | 1.14(0.96,1.35) |
| Grip strength | 1.00 | 1.31(1.14,1.51) |
| Arm muscle quality | 1.00 | 1.25(1.09,1.44) |

HRs were stratified by sex, age, and study regions and shown as point estimate values (95% confidence interval). Models were adjusted for the same variables as model 3 of Table 2 and additionally adjusted for smoking duration.^1^Smoking duration was adjusted as a continuous variable. ^2^According to the AWGS, low muscle mass indices, low grip strength and low arm muscle quality were defined as the lowest sex-specific quintiles of corresponding muscle mass indices, grip strength and arm muscle quality, respectively. Normal groups were defined as Q2-Q5 of corresponding muscle mass indices, grip strength and arm muscle quality, respectively. HRs, hazard ratios.

# Table S5 Sensitivity analyses for associations of muscle mass indices, grip strength, and arm muscle quality with respiratory disease (using Fine-Gray competing risks regression).

| **Muscle mass indices** | **Binary category** | |
| --- | --- | --- |
|  | **Normal**^1^ | **Low**^1^ |
| Total muscle mass index | 1.00 | 1.12(0.96,1.32) |
| Appendicular muscle mass index | 1.00 | 1.04(0.89,1.21) |
| Arm muscle mass index | 1.00 | 1.15(0.99,1.35) |
| Leg muscle mass index | 1.00 | 1.05(0.90,1.23) |
| Trunk muscle mass index | 1.00 | 1.12(0.95,1.31) |
| Grip strength | 1.00 | 1.23(1.07,1.41) |
| Arm muscle quality | 1.00 | 1.16(1.01,1.33) |

HRs were stratified by sex, age, and study regions and shown as point estimate values (95% confidence interval). Models were adjusted for the same variables as model 3 of Table 2. ^1^According to the AWGS, low muscle mass indices, low grip strength and low arm muscle quality were defined as the lowest sex-specific quintiles of corresponding muscle mass indices, grip strength and arm muscle quality, respectively. Normal groups were defined as Q2-Q5 of corresponding muscle mass indices, grip strength and arm muscle quality, respectively. HRs, hazard ratios.

# Table S6 Associations of muscle quality with respiratory disease.

|  | **Binary category** | |
| --- | --- | --- |
|  | **Normal**^1^ | **Low**^1^ |
| **Muscle quality**^2^ | | |
| Cases n | 945 | 401 |
| Rate^3^ | 15.54 | 20.84 |
| Model 1 | 1.00 | 1.37(1.19,1.57) |
| Model 2 | 1.00 | 1.36(1.18,1.56) |
| Model 3 | 1.00 | 1.33(1.15,1.52) |

HRs were stratified by sex, age, and study regions and shown as point estimate values (95% confidence interval). Models 1 to 3 were adjusted for the same variables as the corresponding models in Table 2. ^1^According to the AWGS, low muscle quality was defined as the lowest sex-specific quintiles of muscle quality; normal groups were defined as Q2-Q5 of muscle quality. ^2^Muscle quality was calculated as the ratio of grip strength (kg) to total muscle mass (kg). ^3^Per 1000 person-years; HRs, hazard ratios.

# Table S7 Associations of muscle mass indices, grip strength, and arm muscle quality with respiratory disease by sex.

| **Muscle mass indices** | **Male** | |  | **Female** | | ***P*_interaction_** |
| --- | --- | --- | --- | --- | --- | --- |
|  | **Normal** | **Low** |  | **Normal** | **Low** |  |
| **Total muscle mass index** | | | | | | |
| Cases n | 323 | 113 |  | 700 | 210 |  |
| Rate^1^ | 15.75 | 16.82 |  | 16.43 | 21.12 |  |
| HR (95%CI) | 1.00 | 0.93(0.69,1.27) |  | 1.00 | 1.25(1.02,1.53) | 0.384 |
| **Appendicular muscle mass index** | | | | | | |
| Cases n | 309 | 127 |  | 704 | 206 |  |
| Rate^1^ | 15.80 | 16.54 |  | 16.82 | 19.25 |  |
| HR (95%CI) | 1.00 | 0.96(0.71,1.30) |  | 1.00 | 1.09(0.90,1.33) | 0.750 |
| **Arm muscle mass index** | | | | | | |
| Cases n | 304 | 132 |  | 707 | 203 |  |
| Rate^1^ | 15.42 | 17.49 |  | 16.53 | 20.81 |  |
| HR (95%CI) | 1.00 | 1.06(0.79,1.42) |  | 1.00 | 1.23(1.00,1.50) | 0.901 |
| **Leg muscle mass index** | | | | | | |
| Cases n | 309 | 127 |  | 698 | 212 |  |
| Rate^1^ | 15.72 | 16.76 |  | 16.83 | 19.13 |  |
| HR (95%CI) | 1.00 | 1.02(0.75,1.38) |  | 1.00 | 1.09(0.90,1.32) | 0.890 |
| **Trunk muscle mass index** | | | | | | |
| Cases n | 337 | 99 |  | 715 | 195 |  |
| Rate^1^ | 15.68 | 17.29 |  | 16.55 | 20.92 |  |
| HR (95%CI) | 1.00 | 1.08(0.80,1.47) |  | 1.00 | 1.20(0.98,1.48) | 0.922 |
| **Grip strength** | | | | | | |
| Cases n | 279 | 157 |  | 611 | 299 |  |
| Rate^1^ | 14.32 | 19.94 |  | 16.01 | 20.67 |  |
| HR (95%CI) | 1.00 | 1.31(1.02,1.68) |  | 1.00 | 1.32(1.12,1.57) | 0.248 |
| **Arm muscle quality** | | | | | | |
| Cases n | 315 | 121 |  | 654 | 256 |  |
| Rate^1^ | 14.94 | 19.59 |  | 16.47 | 19.88 |  |
| HR (95%CI) | 1.00 | 1.30(1.02,1.66) |  | 1.00 | 1.23(1.03,1.46) | 0.175 |

HR, hazard ratio; CI, confidence interval.

Models were adjusted for the same variables as model 3 of Table 2, except for sex.

^1^Per 1000 person-years.

# Table S8 Associations of muscle mass indices, grip strength, and arm muscle quality with respiratory disease by age.

| **Muscle mass indices** | **Age <60 years** | |  | **Age ≥60 years** | | ***P*_interaction_** |
| --- | --- | --- | --- | --- | --- | --- |
|  | **Normal** | **Low** |  | **Normal** | **Low** |  |
| **Total muscle mass index** | | | | | | |
| Cases n | 499 | 102 |  | 524 | 221 |  |
| Rate^1^ | 11.69 | 13.60 |  | 23.05 | 27.29 |  |
| HR (95%CI) | 1.00 | 1.19(0.92,1.54) |  | 1.00 | 1.14(0.92,1.42) | 0.901 |
| **Appendicular muscle mass index** | | | | | | |
| Cases n | 502 | 99 |  | 511 | 234 |  |
| Rate^1^ | 11.90 | 12.40 |  | 23.26 | 26.38 |  |
| HR (95%CI) | 1.00 | 1.00(0.77,1.28) |  | 1.00 | 1.10(0.88,1.36) | 0.349 |
| **Arm muscle mass index** | | | | | | |
| Cases n | 505 | 96 |  | 506 | 239 |  |
| Rate^1^ | 11.81 | 12.98 |  | 22.60 | 28.30 |  |
| HR (95%CI) | 1.00 | 1.08(0.83,1.40) |  | 1.00 | 1.23(0.99,1.52) | 0.312 |
| **Leg muscle mass index** | | | | | | |
| Cases n | 500 | 101 |  | 507 | 238 |  |
| Rate^1^ | 11.92 | 12.25 |  | 23.15 | 26.62 |  |
| HR (95%CI) | 1.00 | 0.99(0.77,1.27) |  | 1.00 | 1.15(0.93,1.43) | 0.229 |
| **Trunk muscle mass index** | | | | | | |
| Cases n | 507 | 94 |  | 545 | 200 |  |
| Rate^1^ | 11.73 | 13.54 |  | 23.30 | 26.96 |  |
| HR (95%CI) | 1.00 | 1.20(0.92,1.57) |  | 1.00 | 1.15(0.92,1.43) | 0.855 |
| **Grip strength** | | | | | | |
| Cases n | 516 | 85 |  | 374 | 371 |  |
| Rate^1^ | 11.55 | 15.50 |  | 21.04 | 28.31 |  |
| HR (95%CI) | 1.00 | 1.23(0.96,1.57) |  | 1.00 | 1.35(1.14,1.59) | 0.698 |
| **Arm muscle quality** | | | | | | |
| Cases n | 509 | 92 |  | 460 | 285 |  |
| Rate^1^ | 11.47 | 15.94 |  | 22.70 | 26.97 |  |
| HR (95%CI) | 1.00 | 1.27(1.00,1.62) |  | 1.00 | 1.24(1.04,1.47) | 0.534 |

HR, hazard ratio; CI, confidence interval.

Models were adjusted for the same variables as model 3 of Table 2, except for age.

^1^Per 1000 person-years.

# Table S9 Associations of muscle mass indices, grip strength, and arm muscle quality with respiratory disease by study areas.

| **Muscle mass indices** | **Rural area** | |  | **Urban area** | | ***P*_interaction_** |
| --- | --- | --- | --- | --- | --- | --- |
|  | **Normal** | **Low** |  | **Normal** | **Low** |  |
| **Total muscle mass index** | | | | | | |
| Cases n | 677 | 168 |  | 346 | 155 |  |
| Rate^1^ | 19.00 | 21.26 |  | 12.69 | 16.60 |  |
| HR (95%CI) | 1.00 | 1.09(0.88,1.35) |  | 1.00 | 1.19(0.91,1.56) | 0.550 |
| **Appendicular muscle mass index** | | | | | | |
| Cases n | 637 | 208 |  | 376 | 125 |  |
| Rate^1^ | 18.92 | 21.08 |  | 13.33 | 14.87 |  |
| HR (95%CI) | 1.00 | 1.13(0.92,1.39) |  | 1.00 | 0.90(0.69,1.18) | 0.489 |
| **Arm muscle mass index** | | | | | | |
| Cases n | 680 | 165 |  | 331 | 170 |  |
| Rate^1^ | 19.14 | 20.62 |  | 12.29 | 17.56 |  |
| HR (95%CI) | 1.00 | 1.03(0.83,1.28) |  | 1.00 | 1.35(1.04,1.75) | 0.143 |
| **Leg muscle mass index** | | | | | | |
| Cases n | 619 | 226 |  | 388 | 13 |  |
| Rate^1^ | 18.65 | 21.79 |  | 13.60 | 14.00 |  |
| HR (95%CI) | 1.00 | 1.22(1.00,1.49) |  | 1.00 | 0.82(0.63,1.08) | 0.144 |
| **Trunk muscle mass index** | | | | | | |
| Cases n | 713 | 132 |  | 339 | 162 |  |
| Rate^1^ | 19.37 | 19.71 |  | 12.35 | 17.72 |  |
| HR (95%CI) | 1.00 | 1.00(0.79,1.25) |  | 1.00 | 1.39(1.06,1.82) | 0.075 |
| **Grip strength** | | | | | | |
| Cases n | 562 | 283 |  | 328 | 173 |  |
| Rate^1^ | 17.30 | 25.26 |  | 12.89 | 15.43 |  |
| HR (95%CI) | 1.00 | 1.43(1.19,1.70) |  | 1.00 | 1.15(0.92,1.44) | 0.111 |
| **Arm muscle quality** | | | | | | |
| Cases n | 607 | 238 |  | 362 | 139 |  |
| Rate^1^ | 17.96 | 24.34 |  | 13.32 | 14.71 |  |
| HR (95%CI) | 1.00 | 1.35(1.12,1.62) |  | 1.00 | 1.12(0.90,1.40) | 0.136 |

HR, hazard ratio; CI, confidence interval.

Models were adjusted for the same variables as model 3 of Table 2, except for study area.

^1^Per 1000 person-years.

# Table S10 Associations of muscle mass indices, grip strength, and arm muscle quality with respiratory disease by levels of physical activities.

| **Muscle mass indices** | **Low level** | |  | **High level** | | ***P*_interaction_** |
| --- | --- | --- | --- | --- | --- | --- |
|  | **Normal** | **Low** |  | **Normal** | **Low** |  |
| **Total muscle mass index** | | | | | | |
| Cases n | 568 | 221 |  | 455 | 102 |  |
| Rate^1^ | 19.05 | 23.91 |  | 13.34 | 14.16 |  |
| HR (95%CI) | 1.00 | 1.26(1.01,1.57) |  | 1.00 | 0.99(0.75,1.30) | 0.632 |
| **Appendicular muscle mass index** | | | | | | |
| Cases n | 553 | 236 |  | 460 | 97 |  |
| Rate^1^ | 19.20 | 22.97 |  | 13.66 | 12.71 |  |
| HR (95%CI) | 1.00 | 1.26(1.02,1.57) |  | 1.00 | 0.83(0.63,1.09) | 0.099 |
| **Arm muscle mass index** | | | | | | |
| Cases n | 558 | 231 |  | 453 | 104 |  |
| Rate^1^ | 18.87 | 24.31 |  | 13.33 | 14.16 |  |
| HR (95%CI) | 1.00 | 1.34(1.08,1.67) |  | 1.00 | 0.97(0.74,1.28) | 0.526 |
| **Leg muscle mass index** | | | | | | |
| Cases n | 555 | 234 |  | 452 | 105 |  |
| Rate^1^ | 19.40 | 22.36 |  | 13.48 | 13.48 |  |
| HR (95%CI) | 1.00 | 1.22(0.99,1.51) |  | 1.00 | 0.91(0.70,1.19) | 0.256 |
| **Trunk muscle mass index** | | | | | | |
| Cases n | 601 | 188 |  | 451 | 106 |  |
| Rate^1^ | 19.56 | 22.64 |  | 13.05 | 15.70 |  |
| HR (95%CI) | 1.00 | 1.12(0.90,1.41) |  | 1.00 | 1.13(0.86,1.49) | 0.281 |
| **Grip strength** | | | | | | |
| Cases n | 458 | 331 |  | 432 | 125 |  |
| Rate^1^ | 17.93 | 24.31 |  | 12.75 | 16.72 |  |
| HR (95%CI) | 1.00 | 1.33(1.11,1.58) |  | 1.00 | 1.30(1.02,1.65) | 0.438 |
| **Arm muscle quality** | | | | | | |
| Cases n | 513 | 276 |  | 456 | 101 |  |
| Rate^1^ | 18.65 | 23.84 |  | 13.11 | 15.42 |  |
| HR (95%CI) | 1.00 | 1.28(1.08,1.53) |  | 1.00 | 1.26(0.99,1.62) | 0.808 |

HR, hazard ratio; CI, confidence interval.

Models were adjusted for the same variables as model 3 of Table 2, except for physical activity.

^1^Per 1000 person-years.

# Table S11 Associations of muscle mass indices, grip strength, and arm muscle quality with respiratory disease by BMI.

| **Muscle mass indices** | **BMI < 24kg/m^2^** | |  | **BMI ≥ 24kg/m^2^** | | ***P*_interaction_** |
| --- | --- | --- | --- | --- | --- | --- |
|  | **Normal** | **Low** |  | **Normal** | **Low** |  |
| **Total muscle mass index** | | | | | | |
| Cases n | 366 | 301 |  | 657 | 22 |  |
| Rate^1^ | 16.92 | 20.60 |  | 15.44 | 18.64 |  |
| HR (95%CI) | 1.00 | 1.24(1.04,1.48) |  | 1.00 | 1.12(0.68,1.85) | 0.987 |
| **Appendicular muscle mass index** | | | | | | |
| Cases n | 359 | 308 |  | 654 | 25 |  |
| Rate^1^ | 17.72 | 19.27 |  | 15.42 | 19.22 |  |
| HR (95%CI) | 1.00 | 1.14(0.96,1.36) |  | 1.00 | 1.38(0.87,2.17) | 0.453 |
| **Arm muscle mass index** | | | | | | |
| Cases n | 370 | 297 |  | 641 | 38 |  |
| Rate^1^ | 16.99 | 20.52 |  | 15.30 | 20.96 |  |
| HR (95%CI) | 1.00 | 1.24(1.03,1.48) |  | 1.00 | 1.19(0.79,1.79) | 0.495 |
| **Leg muscle mass index** | | | | | | |
| Cases n | 351 | 316 |  | 656 | 23 |  |
| Rate^1^ | 17.44 | 19.59 |  | 15.53 | 15.70 |  |
| HR (95%CI) | 1.00 | 1.18(0.99,1.40) |  | 1.00 | 1.08(0.68,1.72) | 0.952 |
| **Trunk muscle mass index** | | | | | | |
| Cases n | 422 | 245 |  | 630 | 49 |  |
| Rate^1^ | 17.36 | 20.58 |  | 15.36 | 18.14 |  |
| HR (95%CI) | 1.00 | 1.22(1.00,1.48) |  | 1.00 | 1.12(0.76,1.65) | 0.781 |
| **Grip strength** | | | | | | |
| Cases n | 433 | 234 |  | 457 | 222 |  |
| Rate^1^ | 17.28 | 20.80 |  | 13.86 | 20.44 |  |
| HR (95%CI) | 1.00 | 1.20(0.99,1.48) |  | 1.00 | 1.41(1.15,1.72) | 0.079 |
| **Arm muscle quality** | | | | | | |
| Cases n | 528 | 139 |  | 441 | 238 |  |
| Rate^1^ | 17.76 | 21.23 |  | 14.31 | 18.36 |  |
| HR (95%CI) | 1.00 | 1.24(1.03,1.49) |  | 1.00 | 1.19(0.95,1.50) | 0.414 |

HR, hazard ratio; CI, confidence interval.

Models were adjusted for the same variables as model 3 of Table 2, except for BMI.

^1^Per 1000 person-years.

# Table S12 Associations of muscle mass indices, grip strength, and arm muscle quality with respiratory disease by waist circumference (WC).

| **Muscle mass indices** | **WC < 85(male)/80(female)** | |  | **WC ≥ 85(male)/80(female)** | | ***P*_interaction_** |
| --- | --- | --- | --- | --- | --- | --- |
|  | **Normal** | **Low** |  | **Normal** | **Low** |  |
| **Total muscle mass index** | | | | | | |
| Cases n | 271 | 242 |  | 752 | 81 |  |
| Rate^1^ | 15.45 | 20.80 |  | 16.29 | 17.66 |  |
| HR (95%CI) | 1.00 | 1.28(1.02,1.59) |  | 1.00 | 0.96(0.73,1.28) | 0.364 |
| **Appendicular muscle mass index** | | | | | | |
| Cases n | 266 | 247 |  | 747 | 86 |  |
| Rate^1^ | 16.28 | 19.24 |  | 16.32 | 17.26 |  |
| HR (95%CI) | 1.00 | 1.14(0.92,1.41) |  | 1.00 | 0.93(0.70,1.23) | 0.824 |
| **Arm muscle mass index** | | | | | | |
| Cases n | 270 | 243 |  | 741 | 92 |  |
| Rate^1^ | 15.79 | 20.13 |  | 16.05 | 20.01 |  |
| HR (95%CI) | 1.00 | 1.18(0.95,1.47) |  | 1.00 | 1.13(0.86,1.48) | 0.492 |
| **Leg muscle mass index** | | | | | | |
| Cases n | 269 | 244 |  | 738 | 95 |  |
| Rate^1^ | 16.40 | 19.11 |  | 16.26 | 17.63 |  |
| HR (95%CI) | 1.00 | 1.11(0.90,1.38) |  | 1.00 | 0.98(0.75,1.28) | 0.944 |
| **Trunk muscle mass index** | | | | | | |
| Cases n | 314 | 199 |  | 738 | 95 |  |
| Rate^1^ | 16.12 | 20.63 |  | 16.19 | 18.39 |  |
| HR (95%CI) | 1.00 | 1.20(0.95,1.52) |  | 1.00 | 1.05(0.79,1.38) | 0.715 |
| **Grip strength** | | | | | | |
| Cases n | 333 | 180 |  | 557 | 276 |  |
| Rate^1^ | 16.26 | 20.60 |  | 14.87 | 20.57 |  |
| HR (95%CI) | 1.00 | 1.22(0.97,1.54) |  | 1.00 | 1.36(1.14,1.62) | 0.320 |
| **Arm muscle quality** | | | | | | |
| Cases n | 415 | 98 |  | 554 | 279 |  |
| Rate^1^ | 16.93 | 21.00 |  | 15.33 | 19.00 |  |
| HR (95%CI) | 1.00 | 1.24(1.05,1.47) |  | 1.00 | 1.27(0.98,1.66) | 0.888 |

HR, hazard ratio; CI, confidence interval.

Models were adjusted for the same variables as model 3 of Table 2, except for WC.

^1^Per 1000 person-years.
